# Potential Involvement of *BIRC5* in Maintaining Pluripotency and Cell Differentiation of Human Stem Cells

**DOI:** 10.1155/2019/8727925

**Published:** 2019-01-10

**Authors:** Paulina Gil-Kulik, Arkadiusz Krzyżanowski, Ewa Dudzińska, Jolanta Karwat, Piotr Chomik, Małgorzata Świstowska, Adrianna Kondracka, Anna Kwaśniewska, Maria Cioch, Mariusz Jojczuk, Adam Nogalski, Janusz Kocki

**Affiliations:** ^1^Department of Clinical Genetics, Medical University of Lublin, Lublin, Poland; ^2^Department of Obstetrics and Pathology of Pregnancy, Medical University of Lublin, Lublin, Poland; ^3^Department of Public Health, Faculty of Health Sciences, Medical University of Lublin, Lublin, Poland; ^4^Chair and Department of Haematooncology and Bone Marrow Transplantation, Medical University of Lublin, Lublin, Poland; ^5^Chair and Department of Trauma Surgery and Emergency Medicine, Medical University of Lublin, Lublin, Poland

## Abstract

The *BIRC5* gene encodes a survivin protein belonging to class III of inhibitors of apoptosis, IAP. This protein serves a dual role. First, it regulates cell death, and second, it is an important regulator of mitosis progression, although its physiological regulatory function has not been fully understood. Many studies have shown and confirmed that survivin is practically absent in mature tissues in nature, while its overexpression has been reported in many cancerous tissues. There is little information about the significance of BIRC5 expression in normal adult human stem cells. This paper presents the study and analysis of survivin expression at the transcription level using qPCR method, in hematopoietic stem cells from peripheral blood mobilized with a granulocyte growth factor, adherent cells derived from the umbilical cord, and normal bone marrow stem cells. The expression of this gene was also examined in the blood of normal healthy individuals. The results of the analysis have shown that the more mature the cells are, the lower the expression of the *BIRC5* gene is. The lowest expression has been found in peripheral blood cells, while the highest in normal bone marrow cells. The more the CD34+ and CD105 cells in the tested material are, the higher the *BIRC5* expression is. Stem cells from cell culture show higher *BIRC5* expression. The study confirms the involvement of *BIRC5* from the IAP family in many physiological processes apart from apoptosis inhibition. The possible effect of *BIRC5* on cell proliferation; involvement in cell cycle, cell differentiation, survival, and maintenance of stem cells; and the possible effect of IAP on the antineoplastic properties of mesenchymal stem cells have been demonstrated. Our research suggests that *BIRC5* may be responsible for the condition of stem cell pluripotency and its high expression may also be responsible for the dedifferentiation of tumor cells.

## 1. Introduction

Inhibitors of apoptosis (IAP) are a family of proteins and genes whose primary function is to block cell death in response to a variety of stimuli. Eight proteins from the IAP family (NAIP, cIAP1, cIAP2, XIAP, survivin, BRUCE, ML-IAP, and ILP2) have been identified in humans. They interact with many factors, including the ability to regulate and directly bind caspases, whose activation is inevitable in the correct process of apoptosis. Many human types of cancer have been reported to have increased expression of genes and proteins in the IAP family, in many cases having a negative correlation with the clinical condition of the patient, which in turn makes them an attractive target for antineoplastic therapy. The role of IAP proteins and their physiological functions are not fully understood. It is suggested that, apart from their involvement in pathways of apoptosis, they also play their role in cell differentiation, proliferation, signaling, and immune response [1–3].

Due to numerous studies confirming overexpression of IAP in neoplastic diseases and the frequent occurrence of correlated expression of these genes with unfavorable prognosis, they constitute a potential therapeutic target [4, 5]. An increased expression of inhibitors of apoptosis (IAP) has been reported, among others, in hematological malignancies [6–11], breast cancer [12], colon cancer [13–15], stomach cancer [15, 16], lymphoma, hepatocellular carcinoma [17], head and neck cancer [18], bladder cancer [19], and others. Much attention is also devoted to the possibility of using some IAP as diagnostic and prognostic markers in neoplastic diseases [20, 21]. It has been shown that in some types of cancer, cIAP1, cIAP2, Survivin, and XIAP expression levels are associated with unfavorable prognosis. IAP affect tumor cell activity, their invasion, and metastasis [22]; they are also often responsible for cancer cell resistance to chemotherapy and radiotherapy [1, 7]. In recent years, there have been reports of cancer cell apoptosis induced as a result of selective inhibition of IAP proteins by synthetic particles that act analogously to IAP which destabilize their activity and cause degradation through autoubiquitination [23–26].

Survivin encoded by the *BIRC5* gene is located on 17q25. Survivin is the smallest protein of the IAP family and is 16.5 kDa large. It contains only one BIR domain which is important for its antiapoptotic function, while its CC domain interacts with the tubulin structure. The highest survivin expression was demonstrated in the G2/M phase of the cell cycle, whereas in the G1 phase, there is a rapid decline in its activity. The survivin gene *BIRC5* encodes many genetic variants with unique functions and features, including survivin, survivin-ΔEx-3, survivin-2B, survivin-3B, and survivin 2 alpha. The BIRC5 protein plays a dual role. First, it regulates cell death through indirect or direct interaction with caspases [27], and second, it is an important regulator of mitosis progression and is a component of the CPC complex. It is suggested that survivin, apart from its involvement in cell proliferation [28], plays an important role in cell migration, angiogenesis, DNA damage repair, tissue response to injury, and immune response. In addition, survivin has been shown to regulate the synthesis of microRNA in human leukocytes by limiting the expression of microRNA biosynthesis-controlling proteins at a posttranscriptional level [29]. Most types of cancer are characterized by overexpression of BIRC5 [30]; they include the following types of cancer: breast, liver, ovarian, bladder, lung, stomach, and esophageal and hematological malignancies. In cancer cells, survivin has two main functions; first, it regulates mitosis by forming a CPC complex, and second, it inhibits the process of tumor cell apoptosis. Survivin is one of many important genes that affect tumor aggressiveness and its resistance to therapy, that is why there are numerous attempts to use it in cancer therapy [31–33].

Reactive oxygen species (ROS) leading to the occurrence of oxidative stress have the ability to regulate the expression of genes involved in many processes, among others, immune response or inflammation; it has been shown that oxidative stress can regulate the level of survivin in cancer cells [34, 35]. Survivin overexpression allows survival of cells exposed to oxidative stress [36]. In addition to cancer, increased expression of survivin has been demonstrated in autoimmune diseases and in patients with atherosclerosis in blood lymphocytes [37] which can be connected with increased ROS levels.

Aberrant expression of survivin and disruption of p53 are commonly associated with tumorigenesis [38]. Tumor suppressor gene TP53 regulates expression of genes involved in numerous biological processes, such as the control of the cell cycle, DNA repair, and apoptosis [39]. The tumor suppressor p53 can block cell cycle progression and induce apoptosis. There exists a high possibility that survivin is functionally linked with p53. Many studies have indicated that wildtype p53 can suppress survivin expression at the transcriptional level and that survivin loss of function partially mediates the p53-dependent apoptotic pathway. On the other hand, survivin can regulate p53 expression. One of the studies has shown that overexpression of survivin in human lung cancer cells blocked p53-dependent apoptosis [38].

Overexpression of survivin in carcinoma may overcome cell cycle checkpoints leading to aberrant progression of transformed cells through mitosis [31]. In recent years, efforts have been made to implement survivin as a preferential target and an important prognostic marker in cancer therapy. These approaches based on targeting survivin to enhance tumor cell response to apoptosis and inhibit tumor growth have been developed [38]. It is believed that in some types of cancer, survivin is a diagnostic marker in the early stages [40].

In recent years, numerous studies have been carried out to reduce the expression of the *BIRC5* gene or reduce the protein concentration of survivin in cancer cells and their results are promising. Strategies are tested based on the use of antisense strategy, small interfering RNA, small-molecule survivin inhibitors such as YM 155, or immunotherapy [27, 40].

The greatest hope is placed on survivin-directed therapy due to the fact that it is practically absent in mature tissues in nature, while its overexpression has been reported in many cancerous tissues [41–43].

The aim of our study is to demonstrate the potential role of the BIRC5 gene in maintaining pluripotency and differentiation of human stem cells.

## 2. Material and Methods

The tested material consisted of stem cells and normal cells collected from a group of 131 patients; among them, 43 patients are from the Chair and Department of Haematooncology and Bone Marrow Transplantation, Independent Public Clinical Hospital No. 1 in Lublin, hospitalized between 2014 and 2016, in remission who had previously suffered from hematologic illnesses and provided samples of hematopoietic stem cells from peripheral blood which underwent mobilization through granulocyte growth factor sampled using leukapheresis method. 60 patients are from the Department of Obstetrics and Pathology of Pregnancy, Independent Public Clinical Hospital No. 1 in Lublin, hospitalized between 2014 and 2016, and provided samples of umbilical cords. Cells were isolated from Wharton's jelly by enzymatic extraction (21 patients) and explant method (39 patients). 8 patients are from the Department of Trauma Surgery and Emergency Medicine, Medical University of Lublin, hospitalized between 2014 and 2016, and provided samples of normal bone marrow during hip replacement procedure. 20 healthy volunteers provided samples of peripheral blood. The research was carried out according to the protocol and with the approval of the Bioethics Committee of the Medical University of Lublin, no. KE-0254/128/2014.

Normal cells from peripheral blood and bone marrow were isolated by density-gradient centrifugation using Histopaque-1077 reagent (Sigma, USA) and PBS (BioMed, Lublin, Poland).

Hematopoietic stem cells were isolated in the Department of Haematooncology and Bone Marrow Transplantation, Medical University of Lublin, from the blood of stem cell donors after remission (for autologous transplant). Cells were obtained from peripheral blood, using a cell separator after mobilization with a granulocyte growth factor [44].

Isolation of stem cells from Wharton's jelly from the umbilical cord (from 21 patients-WJC group) was performed by enzymatic digestion using the enzyme collagenase type I (Sigma, USA) [45]. Some of the stem cells from Wharton's jelly (from 39 patients) were isolated using the explant method. The cut pieces of the umbilical cord were placed directly in the culture vessel and incubated for 20 minutes so that the umbilical cord fragments stuck to the plastic surface. Subsequently, the fragments were gently immersed in the growth medium and cultured for 10 days (adherent culture: culture vessel—bottom surface area: 25cm^2^, TC Flask T25, Cell+, (Sarstedt Germany); volume of growth medium—10 ml, composition: DMEM (1x) + GlutaMAXTM-I [+] 1 g/l D-glucose [+] pyruvate (Gibco, UK); heat-inactivated FBS (Gibco, USA); and amphotericin B 250 *μ*g/ml + penicillin/streptomycin (100x) (PAA, Austria)). Culture conditions are as follows: temperature—37°C, O_2_ concentration—4%, CO_2_—5%, and humidity—95%. After approximately 5 days, single cells that migrated from the umbilical cord were observed, sticking to the surface of the culture vessel.

Total RNA was isolated from cells by a modified method of Chomczyński and Sacchi using the following reagents: TRI Reagent (Sigma, USA), chloroform (Sigma, USA), isopropyl alcohol (Sigma, USA), and ethyl alcohol (POSCH).

After isolation, the RNA was dissolved in ultrapure water and the absorbance was measured using a NanoDrop 2000c Thermo Fisher Scientific spectrophotometer at 230 nm, 260 nm, 280 nm, and 320 nm wavelengths. Qualitative and quantitative evaluation of RNA extraction was performed.

Later, the reaction of reverse transcription (RT) was performed using a set of reagents—High-Capacity cDNA Transcription Kits (Applied Biosystems, USA) with RNAz inhibitor (RNase Inhibitor, Applied Biosystems, USA). 1 *μ*g of isolated RNA was used in the RT reaction. The reaction was performed according to the manufacturer's protocol.

The study of *BIRC5* expression was performed at the transcription level using PCR method in real time with StepOnePlus Applied Biosystems. Based on cDNA, qPCR reaction was performed using commercially available reagent kits from Applied Biosystems. TaqMan molecular probes were used for the tested genes: *BIRC5* gene—Hs00153353_m1 (Applied Biosystems, USA), reference gene *GAPDH*—Hs99999905_m1 (Applied Biosystems, USA). The reactions were performed in 96-well plates (MicroAmp Fast Optical 96-Well Reaction Plate, Applied Biosystems, USA) in a volume of 25 *μ*l/well, consisting of 1 *μ*l cDNA synthesized in the reverse transcription reaction, 10.25 *μ*l RNAz- and DNAz-free ultrapure water, 1.25 *μ*l gene-specific probe, and 12.5 *μ*l TaqMan Gene Expression Master Mix (Applied Biosystems, USA). The real-time PCR reaction, after the initial 10-minute denaturation at 95°C, was carried out according to the following scheme—40 cycles: 15 seconds at 95°C and 60 seconds at 60°C.

Gene expression analysis and statistical analysis were performed using ExpressionSuite Software v1.0.3., StepOne Software v2.2.2, and Statistica v13. Relative gene expression (RQ) was calculated by the formula: RQ = 2^−*∆∆*Ct^ [46]. Statistical analysis was performed using one-way ANOVA and Unequal N HSD for post hoc tests. Significant results were recognized for *p* < 0.05.

Some of the collected cells were used for cytometric analysis in order to mark expression of surface antigen characteristic of mesenchymal and hematopoietic stem cells. The research was performed on cells freshly collected from the tested material or after a period of freezing at the temperature of liquid nitrogen (in the case of hematopoietic stem cells isolated from peripheral blood). The percentage of cells demonstrating expression of surface antigens CD34, CD90, and CD105 was evaluated. The cytometric analysis was performed according to the protocol described in our paper [45]. Based on the cytometric analysis, the presence of CD34+, CD105+, and CD90+ cells in the tested material has been confirmed. It has been demonstrated that in the group of cells derived from Wharton's jelly, before and after subjecting to cell culture, more than half of the study population consisted of CD105+ cells. In turn, in the group of mobilized cells isolated from peripheral blood, before and after subjecting to cell culture, CD34+ cells represented the majority of the tested population. In these groups the percentage of CD90+ and CD105+ cells have not exceeded 5%.

## 3. Results

**Figure 1:**
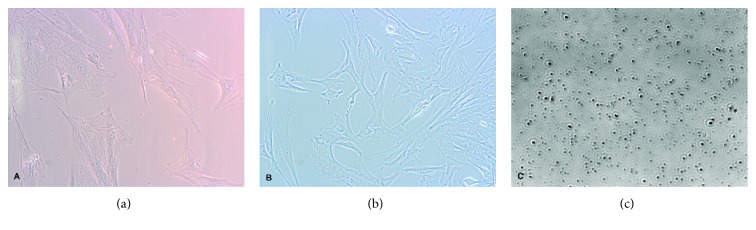
(a, b) Microscopic image of the sample from Wharton's jelly (WJC-CC) blood stem cells (PBSC-CC). The photographs were taken on day 10 during cell culture using an Olympus CKX 41 inverted microscope and Olympus XC50 camera (magnification 200x, eyepiece 10x, and lens 20x). (c) Microscopic image of the sample from peripheral. The photograph was taken on day 10 during cell culture using real-time live cell imaging system xCellence RT and Olympus IX81 inverted microscope (magnification 100x, eyepiece 10x, and lens 10x).

**Figure 2:**
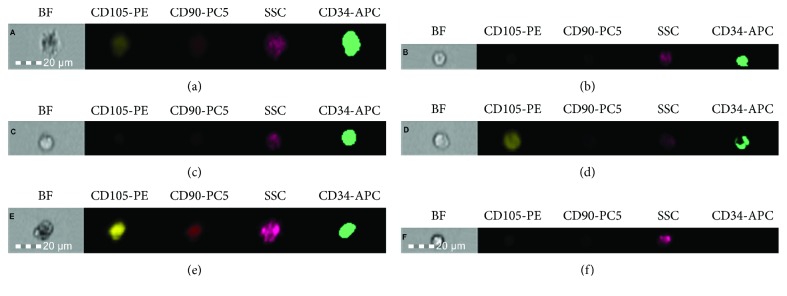
Photographs of single samples of cells from the (a) BM group, (b) PBSC group, (c) PBSC-CC group, (d) WJC group, (e) WJC-CC group, and (f) PBMC group. Photographs presenting BF (microscope image) and fluorescence in channels, showing the expression of studied antigens (CD105, CD90, and CD34). SSC (side scatter—assessment of cell morphology). The analysis and photographs were taken using the FlowSight f. Amnis flow cytometer.

Photographic documentation of the tested cells was performed during cell culture (Figure 1) and during cytometric analysis (Figure 2).

**Figure 3:**
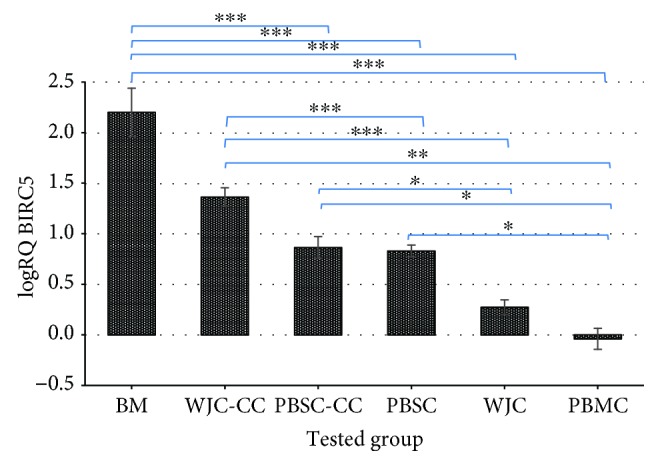
Average *BIRC5* expression on the mRNA level in the group of normal bone marrow cells (BM), peripheral blood hematopoietic stem cells previously subjected to mobilization (PBSC), and Wharton's jelly cells from the umbilical cord isolated using enzymatic digestion with the enzyme collagenase type I (WJC), explant method (WJC-CC), as compared to the average *BIRC5* expression in the group of normal peripheral blood cells from healthy individuals (PBMC) (blood—calibrator). The presented result is shown in the logarithm of average relative expression (logRQ) in the whole tested group ± SE. ^∗∗∗^*p* < 0.0001, ^∗∗^*p* < 0.001, and ^∗^*p* < 0.05.

The research has shown that *BIRC5* demonstrates expression at the transcription level in all trials, in the umbilical cord mesenchymal stem cells, hematopoietic stem cells derived from bone marrow and peripheral blood, and normal blood cells.  Figure 3 shows average relative expression of *BIRC5* (logRQ ± SE) in the tested groups.

Based on the analysis of *BIRC5* expression in the group of tested cells, the lowest average expression has been found in the normal blood cells of healthy individuals, whereas the highest *BIRC5* expression has been demonstrated in the normal bone marrow cells. This group shows nearly 350-fold increase in expression relative to the peripheral blood cells from healthy individuals. *BIRC5* demonstrates expression 59 times significantly higher in cells isolated from the umbilical cord using the explant method, but in cells directly isolated from the umbilical cord using enzymatic digestion, *BIRC5* expression was more than twice higher relative to blood cells. In peripheral blood hematopoietic stem cells subjected to mobilization, *survivin* gene expression is more than 10-fold higher than that of normal mature blood cells (Table 1).

## 4. Discussion

In the research carried out by Fukuda et al. [47], it was noticed that stimulation with a growth factor causes the increase in survivin expression prior to the entry into the cell cycle, which suggests that the increase in survivin expression in hematopoietic stem cells may be related to a regulating influence of growth factor and not only to a cell cycle phase [47]. In our study, higher *BIRC5* expression has been found in cells after 10 days of cell culture, which confirms the contribution of *BIRC5* in a cell cycle and its involvement in cell proliferation. In the case of cultured Wharton's jelly cells, a significantly higher difference in *BIRC5* expression has been observed compared to noncultured cells. It might be caused not only by the involvement of *BIRC5* in cell proliferation but also by a higher content of mesenchymal stem cells in the tested material after adherent cell culture as compared to the noncultured heterogeneous population subjected to enzymatic isolation, which would explain high *BIRC5* expression in stem cells.

The study by Zwerts et al. proved that the structures of the embryo show high survivin expression, whereas its absence in endothelial cells contributes to the death of the embryo. Studies carried out demonstrate that the presence of survivin is essential for normal development and organogenesis. The involvement of survivin in regulation of endothelial cell survival and its influence on maintaining vascular integrity play a vital role in neurogenesis, angiogenesis, and cardiogenesis [48].

Lee et al. in their research speculated that survival of undifferentiated human pluripotent stem cells (hPSC) is highly dependent on antiapoptotic factors like survivin; moreover, they found that exposure of human pluripotent stem cells to a survivin inhibitor like YM155 eliminates undifferentiated hPSC without affecting differentiated cells [49]. Zhou et al was noticed that survivin was one of the factors to participate in human embryonic stem cells or induced pluripotent stem cell pluripotency maintenance. They found that overexpression of survivin in human neural progenitor cells could enhance the efficiency of 1F-OCT4 reprogramming to induced pluripotent stem cells. Authors reported that the mechanism of survivin to maintain the pluripotent state of embryonic stem cells was that it could interact with *β*-catenin in the WNT signal pathway [50]. The research of Kapinas et al. also confirms the commitment of survivin to maintain the state of pluripotency; the authors suggest that survivin is regulated by miR203 and 0 and NANOG [51]. In our study, the correlation between higher *BIRC5* expression and lower cell differentiation status has been proved. This phenomenon may be explained by the involvement of *BIRC5* on the transcription level in maintaining stem cell pluripotency.

Alteri and Mull et al. reported very high levels of survivin expression in embryonic stem cells, pluripotent cells, and somatic stem cells. The authors suggest that survivin expression in these cells is related to regulation of cell proliferation and that it affects the signaling pathway characteristic for stem cells. It is also suggested that survivin in stem cells may be involved in pluripotency control at the level of gene expression, although this function has not been investigated yet [52, 53].

Leung et al. also report high survivin expression in hematopoietic stem cells. The authors suggest that *BIRC5* is a key gene responsible for proliferation and sustaining hematopoietic stem cells and progenitor cells. It was also observed that a decrease in the level of survivin may result in the decrease of cell cycle efficiency, which in turn leads to defects in erythroid maturation. Another research shows that deletion of survivin in mice results in the loss of progenitor cells and hematopoietic stem cells and causes bone marrow ablation and sudden death [54]. In our research, the highest *BIRC5* expression has been found in cells derived from normal bone marrow. Such high *BIRC5* expression is probably due to the fact that bone marrow is a source of both hematopoietic stem cells and mesenchymal stem cells [55, 56].

Conducted own studies and analysis of available literature show that both hematopoietic and mesenchymal stem cells demonstrate survivin expression and that this gene is involved in stem cell survival. It is also essential for their differentiation and proper functioning.

Taking into consideration that many human types of cancer have been reported to have higher expression of genes and proteins from the IAP family which in numerous cases has a negative correlation with the clinical condition of the patient, IAP make an attractive target for antineoplastic therapy. Survivin expression has been reported in many types of cancer, and its level correlates with the more aggressive disease course and poor course of treatment. Normal mature tissues do not demonstrate survivin expression, or it is minimal as confirmed by the study. That is why survivin seems to be both a good diagnostic factor and a good prognostic factor as well as a target for antineoplastic therapy [31].

Fong et al. studied expression of numerous genes, including *BIRC5* at the transcription level in human stem cell collected from Wharton's jelly (hWJSCs). Their analysis confirmed mRNA expression of *BIRC5* in the tested cells. They showed that hWJSC is characterized by lower *BIRC5* expression as compared to embryonic stem cells (ESCs). The authors suggest that lower *BIRC5* expression in hWJSCs as compared to ECSs may be responsible for the antineoplastic properties of mesenchymal stem cells from Wharton's jelly [57]. In our own studies, *survivin* gene expression in mesenchymal cells from the umbilical cord subjected to cell culture (WJC-CC) demonstrates significantly higher levels than those in hematopoietic PBSC and in normal PBMC. On the other hand, cell population derived from the umbilical cord (WJC) directly after collagenase digestion in comparison with hematopoietic blood stem cells demonstrate lower expression, which might be due to the antineoplastic properties of mesenchymal stem cells freshly derived from the umbilical cord.

Lower *BIRC5* expression in mesenchymal stem cells confirms their antineoplastic effect. Increased *BIRC5* expression in hematopoietic stem cells relative to mesenchymal stem cells may be associated with a higher degree of differentiation of these cells which would confirm the involvement of *BIRC5* in cell differentiation.

## 5. Conclusions

The study confirms the involvement of *BIRC5* from the IAP family in many physiological processes apart from apoptosis inhibition. Our research suggests that *BIRC5* may be responsible for the condition of stem cell pluripotency, and its high expression may also be responsible for the dedifferentiation of tumor cells.

## Figures and Tables

**Table 1:** The difference in *BIRC5* expression levels in bone marrow cells (BM), peripheral blood cells subjected to mobilization (PBSC, PBSC-CC) and Wharton's jelly (WJC, WJC-CC), and in peripheral blood cells (PBMC). One-way ANOVA post hoc test (Unequal N HSD).

The difference in *BIRC5* expression levels			*p* value (one-way ANOVA; Unequal N HSD for post hoc tests)					
Tested group	N	*Mean BIRC5* expression (RQ ± SE)	BM	WJSC-CC	PBCS-CC	PBSC	WJSC	PBMC
BM	8	350.2 ± 165.9		0.104711	0.000070^∗^	0.000067^∗^	0.000029^∗^	0.000029^∗^
WJC-CC	39	59.0 ± 17.1	0.104711		0.152726	0.000734^∗^	0.000029^∗^	0.000030^∗^
PBSC-CC	43	18.0 ± 10.6	0.000070^∗^	0.152726		1.000000	0.028835^∗^	0.009662^∗^
PBSC	43	10.0 ± 1.9	0.000067^∗^	0.000734^∗^	1.000000		0.011196^∗^	0.010301^∗^
WJC	21	2.5 ± 0.40	0.000029^∗^	0.000029^∗^	0.028835^∗^	0.011196^∗^		0.993846
PBMC	20	1.2 ± 0.4	0.000029^∗^	0.000030^∗^	0.009662^∗^	0.010301^∗^	0.993846	

## Data Availability

The data used to support the findings of this study are included within the article and can be applicable from the corresponding author.

## Abbreviations

BM: Bone marrow cells isolated using density-gradient centrifugation method (cells collected from a group of 8 patients)
PBSC: Peripheral blood hematopoietic stem cells previously subjected to mobilization (cells collected from a group of 43 patients)
PBSC-CC: Peripheral blood stem cells previously subjected to mobilization and cultured for the period of 10 days (cells collected from a group of 43 patients)
WJC: Wharton's jelly cells from the umbilical cord isolated using enzymatic digestion with the enzyme collagenase type I (cells collected from a group of 21 patients)
WJC-CC: Wharton's jelly cells from the umbilical cord cultured for the period of 10 days (cells collected from a group of 39 patients)
PBMC: Peripheral blood mononuclear cells isolated using density-gradient centrifugation method (cells collected from a group of 20 healthy volunteers).
